# Who leaves the emergency department without being seen? A public hospital experience in Georgetown, Guyana

**DOI:** 10.1186/1471-227X-13-10

**Published:** 2013-06-21

**Authors:** Kendra P Parekh, Stephan Russ, David A Amsalem, Navindranauth Rambaran, Seth W Wright

**Affiliations:** 1 Department of Emergency Medicine, Vanderbilt University Medical Center, 1313 21st Avenue S., 703 Oxford House, Nashville, TN, 37232-4700, USA; 2 Vanderbilt University, School of Medicine, Nashville, TN, USA; 3 Accident and Emergency Department, Georgetown Public Hospital Corporation, Georgetown, Guyana

**Keywords:** Triage, Emergency Department, Quality Assurance, Emergency Care Systems, Left Without Being Seen

## Abstract

**Background:**

Left without being seen (LWBS) proportions are commonly used as quality control indicators, but little data is available on LWBS proportions in the developing world. This study sought to determine the proportion and characteristics of patients who LWBS from the emergency department (ED) of the main public hospital in Georgetown, Guyana.

**Methods:**

This is a retrospective cross-sectional analysis of an ED quality assurance database. Registration personnel collected demographic information on patients presenting to the ED over a 2-week period in July 2010. Both univariate and multivariate analysis were conducted to determine patient characteristics associated with LWBS.

**Results:**

The LWBS proportion was 5.7%. In univariate analysis, patients 18 or older (OR 1.48, 95%CI 1.03-2.12), presenting during the 4PM-12AM shift (OR 2.15, 95%CI 1.53-3.01), with non-urgent triage classification (OR 1.88, 95%CI 1.76-4.66), with non-traumatic chief complaints (OR 1.70, 95%CI 1.14-2.55), or who were not transferred (OR 2.13, 95%CI 1.00-4.55) had significantly higher odds of LWBS. On multivariate analysis, only patients 18 or older (OR 1.54, 95%CI 1.02-2.33), presenting during the 4PM-12AM shift (OR 2.29, 95%CI 1.54-3.40), and with non-traumatic chief complaints (OR 2.39, 95%CI 1.43-4.02) were found to be significantly associated with LWBS. Sex, residence in the capital city, time to triage, transfer status, use of EMS, and triage classification were not statistically associated with LWBS.

**Conclusions:**

LWBS proportions are used as quality control indicators and this study determined the LWBS proportion at a public hospital in a developing country and some of the patient characteristics associated with LWBS. This can be helpful to develop strategies to decrease LWBS proportions and to assess progress over time.

## Background

Patients who leave emergency departments (EDs) without being seen are common in many hospitals. These patients may represent a safety concern. Some patients who leave without being seen (LWBS) have been shown to have deterioration of their medical condition necessitating admission and even urgent surgery[1-3]. These patients are often dissatisfied and may speak negatively of their experiences, altering their use of health services and potentially their friends’ and family’s use of health services[3-6]. Additionally, those who LWBS often seek care from other sources, potentially using more health care resources[1-3]. Although a study from Ontario, Canada found that patients who LWBS are not at higher risk of short term adverse events, this study was conducted in a developed country with universal healthcare and may not hold true in all settings, particularly in resource-poor settings[7]. Thus, high LWBS rates are often still considered a negative quality control indicator.

The ED is often seen as a safety net for patients with limited access to healthcare. This is true in developed countries and likely to be even more of a factor in low and middle income countries where poverty is more prevalent and access to primary care is often limited. Consequently, leaving without evaluation by a clinician may pose an even greater risk of health deterioration in developing nations. However, there is a relative paucity of data on proportions of LWBS and patient characteristics associated with LWBS in these countries. The majority of published studies originate from EDs in Australia, North America, and the United Kingdom[5,6,8-10]. Notably, recent reviews of LWBS rates and patient characteristics associated with LWBS did not include any data from low or middle income countries[11,12]. As emergency care expands in developing nations, it is important to document LWBS proportions to develop appropriate quality control benchmarks, to measure progress and most importantly to improve patient care in this vulnerable population.

Guyana is a developing country located on the northern coast of South America. It is culturally and economically a Caribbean community. It is considered to have a lower middle income economy and its economic and healthcare indicators lag behind those of most of the surrounding Caribbean and South American countries[13]. Thus, this study sought to determine the proportion and characteristics of patients who LWBS from the ED of the main urban, public hospital in Guyana.

## Methods

### Study design

This study is a retrospective cross-sectional analysis of an ED quality assurance database collected at the Georgetown Public Health Corporation (GPHC) located in Georgetown, Guyana. This quality assurance database was created at the request of GPHC management and the Guyana Ministry of Health to better quantify the demographics of the ED population. Typically, detailed ED patient registration data were not recorded. The quality assurance data were collected during a two-week time period in July 2010. During this time period, dedicated registration personnel prospectively collected detailed patient information as part of a quality assurance survey. Data collection was performed by experienced, full-time ED registration personnel that were specifically trained by a systems analysis consultant for this quality assurance project. Since this study is being performed retrospectively, data collectors were not aware of the study hypothesis. Extensive patient information was obtained. Data collected included: age, sex, geographic area of residence, use of emergency medical services (EMS) transportation, transfer status (i.e. official transfer from another health care facility), time of presentation, time in minutes from arrival to triage, triage score, and chief complaint. The time of arrival for each patient was specifically recorded by the dedicated data collector and was defined as the time the patient arrived through the door of the ED. The data collector also recorded the time each patient was triaged. Data collectors were not instructed to record times that patients left without being seen. The study protocol was reviewed by the Vanderbilt University Institutional Review Board and GPHC provided written approval for use of the database.

### Setting

In Guyana, the majority of healthcare is publicly funded, but there are private clinics and private hospitals available. GPHC is the main teaching hospital in Guyana and serves as both a regional public hospital and as the national referral hospital. Patients are not charged for emergency or routine services at GPHC. The ED has an estimated annual volume of 75,000. Memory-based triage is performed by a registered nurse and consists of a three-level acuity system with patients triaged into immediate, urgent, or non-urgent classifications. There are limited data on the reliability and validity of this system. The triage system is based on physiologic criteria, “red flags” for critical diagnosis, and identification of high risk features (medical comorbidities, immunocompromised status, etc.). The triage nurse sends the majority of patients to an adjacent waiting area after completion of the triage process. However, patients can also be triaged directly to outpatient hospital clinics and these patients do not receive care in the ED. Physicians control most patient flow from the waiting area to the treatment area by requesting the next patient when they are ready for a new patient encounter. Triage nurses can take critically ill patients directly to a patient care area. At the time of study, the ED was staffed with residents and general medical officers; there were no Emergency Medicine trained physicians. An Emergency Medicine training program began at GPHC in October 2010 but was not in existence at the time of the study.

### Data analysis

Patients who were triaged directly to outpatient clinics were excluded from analysis. Age was dichotomized for analysis into less than or greater than 18 years and the geographic area of residence was dichotomized into residence in the capital city of Georgetown versus residence in any location in the country outside of Georgetown. Time of presentation was divided into three time periods consistent with nursing shifts (8AM – 4PM, 4PM – 12AM, 12AM – 8AM). The chief complaint was categorized into traumatic or non-traumatic complaints. An initial univariate comparison was conducted with LWBS as the dependent variable. In the univariate analysis, categorical variables were analyzed using logistic regression and are presented with odds ratios with 95% confidence intervals. The sole continuous variable, time from arrival to triage, was analyzed with the t-test. A p-value of < 0.05 was considered statistically significant. Logistic regression analysis was also used in a multivariate model to determine the odds ratio for each covariate with LWBS as the dependent variable. All available covariates were entered into the multivariate logistic regression model. Listwise deletion was used for each regression to exclude observations with missing data. The number of observations after listwise deletion was 2434. The overall p-value of the model was <0.001 with a likelihood chi-square statistic of 47.51. Odds ratios with 95% confidence intervals are presented for categorical variables along with the p-value. Statistical analysis was done using Stata/MP 12.0 for Mac (StataCorp LP, College Station, TX).

## Results

A total of 3377 patient visits were included in the database. Three-hundred and fifty patients were triaged directly to a hospital clinic and excluded from analysis, leaving 3027 visits for analysis. Overall, 173 patients left the ED after triage and prior to evaluation by a physician. The LWBS proportion was 5.7% (173/3027). For patients with the most acute triage score, 3.1% (5/162) LWBS.

Table 1 compares those who LWBS to those who stayed for evaluation. In this univariate analysis those who were age 18 or older, presented during the 4PM-12AM shift, had a non-urgent triage classification, had a non-traumatic chief complaint, or were not transferred had significantly higher odds of LWBS.

**Table 1:** Comparison of patients who LWBS and those who stayed for evaluation

Variable*	**LWBS**	**Did not LWBS**	**% LWBS**	**Odds Ratio (95% CI)**
	**N = 173 (5.7%)**	**N = 2854 (94.3%)**		
Age				
<18 years old	41	899	4.36%	Reference
≥ 18 years old	132	1955	6.32%	1.48 (95%CI 1.03-2.12)
Sex				
Male	80	1374	5.50%	Reference
Female	89	1473	5.70%	1.04 (95%CI 0.76-1.42)
Region of residence				
Georgetown	134	2277	5.56%	Reference
Other region	39	577	6.33%	1.15 (95%CI 0.79-1.67)
Shift of presentation				
8AM-4PM	57	1372	3.99%	Reference
4PM-12AM	94	1053	8.20%	2.15 (95%CI 1.53-3.01)
12AM-8AM	22	429	4.88%	1.23 (95%CI 0.74-2.04)
Mean time from ED arrival to triage (minutes)	34.2	36.4		p = 0.51
Triage score				
Immediate	5	157	3.09%	Reference
Urgent	27	337	7.42%	2.52 (95%CI 0.95-6.66)
Non-urgent	131	2189	5.65%	1.88 (95%CI 1.76-4.66)
Type of complaint				
Traumatic	30	786	3.68%	Reference
Non-traumatic	134	2060	6.11%	1.70 (95%CI 1.14-2.55)
Transportation				
EMS	5	192	2.54%	Reference
Other	156	2579	5.70%	2.33 (95%CI 0.94-5.88)
Transfer status				
Transferred	7	249	2.73%	Reference
Not transferred	145	2400	5.70%	2.13 (95%CI 1.00-4.55)

*Not all variables were documented for every patient visit.

Multivariate logistic regression analysis (Table 2) demonstrated significantly increased odds of leaving prior to physician evaluation in patients who were 18 years of age or older, presented during the 4PM-12AM shift, and had non-traumatic conditions. Sex, residence within Georgetown, time from presentation to triage, transfer status, and use of EMS transportation were not significantly associated with LWBS on the multivariate analysis. Triage classification was not significantly associated with LWBS but a trend towards significance was noted among those with non-urgent compared to immediate triage classification.

**Table 2:** Results of multivariate analysis

**Variable**	**Odds Ratio**	**95% CI**	**p-value**
Age ≥ 18 years old	1.54	1.02-2.33	0.039
Male sex	1.13	0.79-1.62	0.505
Residence in Georgetown	1.12	0.67-1.90	0.660
Shift of presentation			
4PM-12AM	2.29	1.54-3.40	<0.001
12AM-8AM	1.31	0.71-2.39	0.389
Time from ED arrival to triage	1.00	0.99-1.00	0.431
Triage score			
Urgent	1.70	0.66-4.25	0.273
Non-urgent	2.76	0.98-7.76	0.054
Non-traumatic complaint	2.39	1.43-4.02	0.001
Non-EMS presentation	1.49	0.46-4.81	0.508
Not transferred	1.94	0.68-5.54	0.218

## Discussion

The proportion of patients LWBS after presenting for emergency care varies considerably among hospitals and over time. A national study of patients who LWBS in the United States found an overall LWBS proportion of 1.7%[10], but proportions reported at individual institutions within the United States have ranged from 0.84% to 15%[11,12]. In multiple reviews, the lowest LWBS proportion reported was 0.1% in Taiwan[14] while proportions in Australia have been reported from 1.7% to 8.6%[11,12], proportions in the UK from 3.26% to 7.2%[11,12], and proportions in Canada from 1.4% to 4.5%[11,12]. Although these are the proportions reported in the literature, true proportions may be different. Regardless, data from the developing world are limited but at a public teaching hospital in Trinidad, the proportion of injured patients who LWBS was found to be 11.6%[15]. In this study we found the proportion of patients who LWBS was 5.7% for patients presenting to the major public referral hospital in the capital city of Guyana. The proportion of patients who LWBS at this hospital is high by some international standards but is still well below that seen in many urban public hospital systems in developed countries and below the best available data from other developing countries.

We found increased odds of LWBS among adults compared to pediatric age patients. Others have not noted a lower proportion of LWBS among pediatric patients presenting to general EDs[15,16]. Notably, some pediatric hospitals in North America report extremely high LWBS rates, with some as high as 16.6%[16,18]. The reasons for our finding are unclear but it is possible that pediatric patients seen in the ED in Guyana are sicker than the typical patient in North America or other developed areas and the parents are more likely to remain for care despite a long wait. It has also been noted that pediatric patients who LWBS in North America almost all have primary care providers[19]. It is possible that a real or perceived decreased ability to access primary care would influence the decision of the parents to stay for care. In Guyana, although there is access to primary care, it is often not on an appointment basis and wait times in clinics can be prolonged.

Similar to previous studies, we found that presenting during the second shift (4PM-12AM) was associated with significantly higher odds of LWBS[3,14,17-20]. The fact that the proportion of patients who LWBS differs by time of day is not unexpected given that high LWBS proportions are usually reflective of congestion within the ED and this has been noted in other studies[6,11,16-19,21]. While the day shift (8AM - 4PM) at GPHC has a higher patient volume, the cumulative back up of patients starts during the mid-day time period and continues throughout the later shifts, likely contributing to an increased likelihood of LWBS during that time period. Although the mean time from arrival to triage was not statistically different between patients who waited for care and those who LWBS, this may be secondary to the fact that the decision to LWBS has more to do with longer wait time for care.

We noted increased odds of LWBS in patients with non-traumatic conditions. This finding is expected given that most patients with injuries require acute attention. Transfer from other health care facilities and mode of transportation (EMS vs. other methods) were not associated with statistically significant differences in LWBS on multivariate analysis. The lack of significance is possibly due to low patient numbers among those transferred and those arriving by EMS. In Guyana, EMS is markedly underdeveloped and often is unavailable, even in the setting of critical illness or injury.

In most studies, patients with more acute triage levels have lower rates of LWBS[3,10,12,14,20]. We did not note a statistically significant difference in the proportions of LWBS in this three-level triage system on the multivariate analysis. There was, however, a strong trend toward significance. Lack of significance in this study was likely due to the small numbers of patients triaged to higher acuity levels and possibly to problems with the ability of the triage system in differentiating various levels of care. Although it would seem that those triaged as non-urgent could defer care, studies have found that these patients are potentially sick[1-3]. Notably, 3.1% of the patients with the highest triage scores LWBS in this study. As unexpected as this would seem, other studies have found that patients in the highest triage categories will still LWBS[10,14].

Apart from patient characteristics associated with LWBS, there are numerous hospital-associated factors that make it likely that LWBS proportions would be high in developing countries. Hospital overcrowding is common in many developing countries and overcrowding is well known to lead to prolonged patient wait times[3,4,6,8,12,21]. Not surprisingly, a prolonged wait time is the primary reason cited by patients who LWBS[3,5,12,14]. Adequate clinical space for providing emergency care is a significant problem in many healthcare systems. This is clearly a factor at GPHC where the ED clinical space is limited in comparison to North American hospitals with similar patient volumes. A variety of hospital-related strategies, including use of multiple quality improvement measures[22], addition of a fast-track area[23], addition of mid-level practitioners[24], addition of higher level practitioners at triage[25] and the use of queuing theory[26] have been assessed for changes in LWBS proportions with mostly positive effects. Unfortunately, many of these modalities are not practical in a resource-constrained environment. Nevertheless, GPHC is actively seeking solutions to address this issue and has recently added a physician in triage. During peak hours, this physician is based in the triage area with the goal of identifying those in need of immediate treatment and expediting care for those with minor conditions. Anecdotal reports are positive, but the effect of this staffing plan on ED crowding, waiting times and LWBS proportions has yet to be formally studied.

### Limitations

Although this was a retrospective analysis of a quality assurance database, all data were collected prospectively and an a priori objective of the database was to determine the proportion of patients who LWBS. Thus, the retrospective use of this database was unlikely to have led to significant bias. GPHC had not previously collected detailed patient characteristics at registration. This data was initially collected for quality assurance purposes and GPHC administrators set the time limits on data collection. The database was collected over an isolated two-week time period. Ideally, we would have examined the LWBS proportion over a longer time period in order to eliminate seasonal variation or other causes of variation in LWBS proportions. However, accurate longer-term data regarding patients who LWBS or accurate demographic information are not currently available at GPHC. We excluded patients who were sent from triage directly to a hospital clinic for care. It is possible that following these patients and including them in the analysis would have changed our results. Similarly, patients who presented for care but left prior to triage were not included in this study as no data could be obtained on these patients. Including these patients may have increased the LWBS proportion, but inclusion of these patients would also have made it difficult to compare our results with other published studies. Patients leaving before registration or triage are not typically reported in similar studies. Another limitation of this study was the lack of patient outcomes for those who LWBS. There was no mechanism to determine if patients who LWBS suffered other adverse events such as re-presentation to the ED, hospitalization, procedural interventions, or death. Finally, although this study was conducted at the primary referral hospital in a developing country, it may be difficult to generalize these findings to other health care institutions in Guyana or in other developing countries.

## Conclusions

The proportion of patients who LWBS is often used as a quality control indicator. There is a paucity of data on patients who LWBS in developing countries where those who LWBS may be more vulnerable to poor outcomes. As emergency care expands in developing nations, it is important to document LWBS proportions to develop appropriate quality control benchmarks and measure progress. The LWBS proportion at an urban, public hospital in Guyana, South America was found to be 5.7% with increased odds of LWBS associated with adult patients, presentation during the second shift, and presentation with non-traumatic conditions. This data provides useful information to develop strategies to decrease the number of patients who LWBS and can be followed over time to assess progress.

## Abbreviations

ED: Emergency department; EMS: Emergency medical services; GPHC: Georgetown Public Hospital Corporation; LWBS: Left without being seen.

## Competing interests

The authors have no competing interests to declare.

## Authors’ contributions

KPP participated in data analysis and interpretation and drafted the manuscript. SR participated in study design and data analysis. DA participated in study design and data acquisition. NR participated in study design and critically revised the manuscript. SWW participated in study design, data analysis and interpretation, and critically revised the manuscript. All authors read and approved the final manuscript.

## Pre-publication history

The pre-publication history for this paper can be accessed here:

http://www.biomedcentral.com/1471-227X/13/10/prepub
